# Integration of Next-generation Sequencing and Immune Checkpoint Inhibitors in Targeted Symptom Control and Palliative Care in Solid Tumor Malignancies: A Multidisciplinary Clinician Perspective

**DOI:** 10.7759/cureus.2909

**Published:** 2018-07-02

**Authors:** Doron Feinsilber, Marco Ruiz, Sorin Buga, Leigh A Hatch, Andrew D. Hatch, Katrina A Mears

**Affiliations:** 1 Hematology/Oncology, Medical College of Wisconsin/Froedert Cancer Center, Milwaukee, USA; 2 Memorial Cancer Institute, Memorial Healthcare System, Hollywood, USA; 3 Supportive Care Medicine, City of Hope Medical Center, Duarte, USA; 4 Morsani College of Medicine, University of South Florida, Tampa, USA; 5 National Ophthalmic Research Institute, Retina Consultants of Southwest Florida, Tampa, USA; 6 Ophthalmology, Retina Consultants of Southwest Florida/National Ophthalmic Research Institute, Fort Myers, USA

**Keywords:** targeted therapy, uveal melanoma, palliative, kras, nras, braf, symptom control, metastatic colorectal cancer, pd-1 inhibitor, next generation sequencing

## Abstract

The molecular characterization of solid tumor malignancies with respect to tumorgenesis, risk stratification, and prognostication of chemotherapeutic side effects is multi-faceted. Characterizing these mechanisms requires a detailed understanding of cytogenetics and pharmacology. In addition to the standard palliative care interventions that address issues such as fatigue, neuropathy, performance status, depression, nutrition, cachexia, anxiety, and medical ethics, we must also delve into individual chemotherapy side effects. Comprehending these symptoms is more complex with the advent of broader targeted therapies. With the advent and initiation of Foundation Medicine (FMI) testing, we have been able to tailor regimens to the individual genetics of the patient. Next-generation sequencing (NGS) is a bioinformatic analysis used in order to create a targeted effort to understand the complex genetics of a vast array of malignancies. Through the process known as high-throughput sequencing we, as clinicians, can obtain more real-time genetic data and incorporate the information into our reasoning process. The process involves a broad manner in which deoxyribonucleic acid (DNA) sequence data is obtained including genome sequencing and resequencing, protein-DNA or proteinomics, chromatin immunoprecipitation (ChIP)-sequencing, ribonucleic acid (RNA) sequencing, and epigenomic analysis. High-throughput sequencing techniques including single molecule real-time sequencing, ion semiconductor sequencing, pyrose sequencing, sequencing by synthesis, sequencing by ligation, nanopore sequencing, and chain termination (otherwise known as Sanger sequencing) have expanded the realm of NGS and clinicians options.

## Editorial

As the gap between palliative medicine and solid tumor oncology continue to distinctly converge through greater and broader understanding of receptor chemistry, signal transduction pathways, and unique cytotoxic profiles, both specialties will be able to contribute to a team approach. The understanding and molecular characterization of tumors is possible due to next-generation sequencing (NGS), with novel methods being developed in an attempt to make sequencing more cost-effective and available across distinct fields [1]. NGS is important for personalizing tumor therapies to an individual’s genome, allowing for both the targeting of specific tumor mutations as well as gaining an understanding of what the side effect profile will be for a specific patient. With an increased emphasis on personalized medicine and the integral role an individual’s genome plays in disease states, NGS has never been more essential for patient care [2].

NGS, also referred to as high-throughput sequencing, includes a variety of molecular methods that can be used to assess individual genomic information, comprising chromatin immunoprecipitation (CHiP) sequencing, ribonucleic acid (RNA) sequencing, epigenomic analysis, and resequencing. In CHiP-sequencing, chromatin immunoprecipitation is followed by deoxyribonucleic acid (DNA) sequencing in order to map out the binding sides of DNA-binding proteins, without a hybridization array as is necessary in CHiP-chip technology. With CHiP-seq, genome-wide mapping of protein-DNA interactions is possible, allowing researchers and clinicians to understand critical cellular processes such as transcription, replication, and DNA repair, especially as they pertain to tumor mutations [3]. RNA sequencing is another method that uses NGS to actively assess what RNA is present in cells, effectively representing what portions of the genome are being transcribed. RNA-seq is conducted by isolating RNA, synthesizing complementary deoxyribonucleic acid (cDNA), and then analyzing the cDNA library in order to study gene expression, post-transcriptional modifications, and other information about the transcriptome. Epigenomic analysis specifically analyzes gene expression influenced by the heritable modification of histones and DNA methylation. This is accomplished by genome-wide methylation analysis. Finally, resequencing is another method that uses NGS for sequencing of a specific gene variation found in an individual when a genome of the species is available for reference [4]. All of these methods have incredible utility in the field of oncology and palliative care, as NGS can be used to identify and characterize tumors, as well as identify specific potential side effects of therapy based on an individual’s genome [3].

To illustrate, an understanding of the KRAS biology of our patients with colorectal cancer (CRC) can determine not only response to therapy, but also enhance understanding and probability of common toxicities. Some of these toxicities include the severity of skin rash, hypomagnesemia, neuropathy, mucositis, and sensitivity to 5-fluorourcail (5-FU) based chemotherapy. KRAS is a member of the Ras superfamily of signal transduction proteins that ultimately are responsible for epidermal growth factor receptor (EGFR) signaling activation. When utilizing NGS, mutation types can be characterized and ultimately factored into treatment decisions [4].

Integrating KRAS mutation data with cachexia

Murine models have displayed the presence of KRAS inducing Src-dependent expression of the PEAK1 protein. In-vivo mouse models expressing KRAS G12D constitutively have activated KRAS/Src signaling through Src kinase activation. KRAS-driven tumors with strong constitutive expression can result in worsening cachexia by a variety of defined mechanisms, one being increased macropinocytosis with significant nutrient uptake, including branched chain amino acids. This is one of the several mechanisms that are keenly involved in hypoalbuminemia and subsequent third spacing. With a higher tumor burden and metabolic requirements, mitochondria upregulate the uncoupling protein 1 (UCP-1 protein), effectively shifting utilization of metabolic storage sites from white to brown adipose tissue. When this occurs, a plethora of cachexin cytokines, including interleukin-6 (IL-6), interleukin-1 (IL-1), and tumor necrosis factor alpha (TNF-alpha), compromise skeletal muscle. Increased metabolic rates and elevated cachexin cytokines may also result in hypophosphatemia, which in turn can result in profound weakness, increased aspiration risk, and in more severe cases hemolytic anemia. From a clinical perspective, the loss of defined skeletal muscle can increase fall risk and decrease ambulation, which may contribute to higher risk of intracranial bleed and thrombosis, respectively. Sarcopenia is a genuine concern in patients with metastatic solid tumor malignancies that can effect eastern cooperative oncology group (ECOG) performance status and ultimate response to therapy. Understanding the status of microsatellite instability plays an integral role as well. Typically, microsatellite instability indicates increased CpG islands of methylated promoters that yield a less favorable prognosis and resistance to EGFR targeted therapies [4].

Role of NGS testing and molecular synergism in uveal melanoma

Uveal melanoma accounts for approximately 85% of all ocular melanomas and approximately 50% of these patients develop metastatic disease [5]. In making an appropriate determination as to targeted therapies and clinical responses, NGS testing early on can yield a plethora of information. Ophthalmologists are increasingly aware of targeted therapies and their propensity to cause systemic side effects. A 12-gene panel is utilized in initial molecular characterization to learn more about a specific tumor, particularly data pertaining to autophosphorylation and activation of the PI3K/Akt [LH1] and RAS pathways. Dual kinase inhibition with MEK inhibitors such as trametinib and Akt identification plays an integral role. Determination of BRAF V600E mutations in ocular melanoma allows for greater therapeutic efficacy for BRAF inhibitors such as vemurafinib and dabrafenib. For distant metastasis, particularly involving the central nervous system (CNS), a combination of BRAF and MEK inhibition is of greatest efficacy [2]. Promising data exists for ipilipumab, a CTLA-4 inhibitor. With the invention of programmed cell death protein 1 (PD-1) and programmed death ligand 1 (PDL-1) checkpoint inhibitors as nivolumab and pembrolizumab, some efficacy has been determined in the absence of a viable quantitative receptor assay. Identifying potential side effects including autoimmune hepatitis, cytokine release syndrome, and multiple sclerosis are important when patients are receiving systemic therapy [2].

Uveal melanomas, unlike cutaneous melanomas, rarely contain NRAS, NF1, or BRAF mutations and are more frequently characterized by point mutations in the G-protein α-subunit. The GNAQ and GNA11 genes code for the α-subunit of G proteins that act in conjunction with G-protein-coupled receptors. Mutations in genes associated with GNAQ or GNA11 are reported in over 80% of primary uveal melanomas and are associated with constitutive activation of signaling pathways including the central oncogenic RAS/RAF/MEK/ERK (RAS-ERK) pathway, thereby driving cell proliferation [5].

The immune checkpoint inhibitors with their vast increase in utilization in standard national comprehensive cancer network (NCCN) algorithms in solid tumor malignancies have a more complex physiological mechanism in interfering with both the cell-mediated and humoral immune system. The manner in which immune checkpoint inhibitors pharmacologically perform effect a multitude of organ systems with an incidence up to 90% as mentioned in several studies [3]. Most commonly affected systems are the gastrointestinal tract, skin, respiratory, endocrine, and musculoskeletal systems whereas the cardiovascular, renal, neurologic, ophthalmologic and hematologic systems seem to be less affected [2].

The side effects are graded on a 4-point scale where the least severe grade 1 and 2 are more frequently seen in the cutaneous and gastrointestinal systems whereas the most severe grades 3 and 4 which may require hospitalization are present in the colon and endocrine system. Use of corticosteroids oral or intravenous (iv) is indicated only when the side effects are of grade 2 or greater in which symptoms for the patient begin to be markedly noticeable and affect quality of life. The treatment paradox is that steroids will erode the therapeutic value of the drug. Grade 2 requires oral prednisone or iv methylprednisolone at 0.5-1 mg/kg body weight/day, whereas in grade 3 and 4 a dose of prednisone of 1-2 mg/kg body weight/day or equivalent dose of iv methylprednisolone is used. The steroid taper should be initiated the moment patients experience improvement of their adverse symptoms. From a clinical perspective, such patients on a prolonged steroid taper should be considered for prophylaxis for *Pneumocystis carinii*. The immune-related side effects require a multidisciplinary approach from various disciplines because of the multitude of organs that are affected by them [2].

Careful screening of all patients considered for immunotherapy is recommended in an attempt to avoid or at least minimize these side effects. A detailed analysis should include the patient’s prior history of an autoimmune condition or infectious diseases, bowel habits, skin conditions or endocrine dysfunctionality. It is recommended to routinely order blood tests like: complete blood count (CBC), comprehensive metabolic profile (CMP), thyroid stimulating hormone (TSH), hemoglobin A1c (HgbA1c), creatinine kinase (CK), lipid panel along with an infectious disease screen (hepatitis and human immunodeficiency (HIV) panels). Cardiac tests such an electrocardiogram (EKG) and a baseline and weekly troponin levels for the first six weeks along with a baseline oxygen saturation monitoring are indicated as well. Patients with at high risk of toxicity or with a preexisting condition are indicated to undergo a morning cortisol and adrenocorticotrophic hormone (ACTH) levels, pulmonary function tests, a 6-minute walk test and brain natriuretic peptide (BNP) and natriuretic peptide levels [2].

Many of the patients on immunotherapy will develop pain related either to the cancer itself or to the side effects from cancer therapy with the opioids being an essential tool in pain palliation. Special considerations when choosing an opioid versus other are the presence of opioid tolerance, creatinine clearance, presence of side effects from an opioid formulation that required an opioid rotation, ability to swallow and a history of substance abuse. Long-acting formulations of opioids are indicated only in opioid-tolerant patients where opioid tolerance is defined as taking at least 60 mg oral morphine per day, 25 mcg transdermal fentanyl per hour, 30 mg oral oxycodone per day, 8 mg oral hydromorphone per day, 25 mg oral oxymorphone per day, 60 mg oral hydrocodone per day, or an equal analgesic dose of another opioid for one week or longer [2].

Fentanyl is the preferred option in patients with difficulties swallowing or with renal failure where morphine is absolutely contraindicated due to high risk of opioid-induced neurotoxicity by the accumulation of morphine 3 and 6 glucuronide that are renally cleared. The clinicians must also not forget about the risk of serotonin syndrome when introducing tramadol or tapentadol to patients already on antidepressants, such as selective serotonin reuptake inhibitors (SSRIs), serotonin and norepinephrine reuptake inhibitors (SNRIs), tricyclic antidepressants (TCAs). The iv route for opioid administration, such as boluses or patient-controlled analgesia, needs to be considered when patients have difficulties swallowing or experiencing severe uncontrolled pain that requires a rapid titration to minimize physical suffering. Methadone, a mu receptor agonist, and an N-methyl-D-aspartate (NMDA) receptors antagonist, could also be employed in managing cancer pain preferably under the supervision of a pain management or palliative care specialist, according to NCCN recommendations, due to its unpredictability and multiple side effects. These side effects include cardiac risk, which is minimized by checking a baseline QT interval on an EKG. Particular attention needs to be paid in screening for potential substance use disorders and monitoring compliance with opioid therapy in the context of the rampant opioid crisis we are currently facing in the United States [2].
